# Immunophenotypic analysis on circulating T cells for early diagnosis of lung cancer

**DOI:** 10.1186/s40364-024-00713-7

**Published:** 2024-12-26

**Authors:** Sung-Woo Lee, Young Ju Kim, Kyung Na Rho, Saei Jeong, Jeong Eun Noh, Hee-Ok Kim, Hyun-Ju Cho, Ju Sik Yun, In-Jae Oh, Jae-Ho Cho

**Affiliations:** 1 Department of Microbiology and Immunology, Chonnam National University Medical School, Hwasunup, Jeollanamdo 58128 Republic of Korea; 2 Medical Research Center for Combinatorial Tumor Immunotherapy, Chonnam National University Medical School, Hwasunup, Jeollanamdo Republic of Korea; 3 National Immunotherapy Innovation Center, Chonnam National University Medical School, Hwasunup, Jeollanamdo Republic of Korea; 4 BioMedical Sciences Graduate Program, Chonnam National University Medical School, Hwasunup, Jeollanamdo Republic of Korea; 5 Selecxine Inc, Seoul, Republic of Korea; 6 Department of Internal Medicine, Chonnam National University Medical School, Hwasun Hospital, Hwasunup, Jeollanamdo 58128 Republic of Korea; 7 Department of Thoracic and Cardiovascular Surgery, Chonnam National University Medical School, Hwasun Hospital, Hwasunup, Jeollanamdo Republic of Korea

**Keywords:** Immunophenotyping, Diagnosis, Lung cancer, Liquid biopsy

## Abstract

The immune system continuously interacts with tumors, possibly leading to systemic alterations in circulating immune cells. However, the potential of these cancer-associated changes for diagnostic purposes remains poorly explored. To investigate this, we conducted a comprehensive flow cytometric analysis of 452 peripheral blood mononuclear cell (PBMC) samples from 206 non-small-cell lung cancer (NSCLC) patients, 100 small-cell lung cancer (SCLC) patients, 94 healthy individuals, and 52 benign lung disease (BLD) patients. We focused specifically on circulating T cells, given their close interaction with tumors, and initially assessed 93 T-cell features from the flow cytometric analysis. Using a feature selection protocol, we identified five T-cell features in peripheral blood with strong diagnostic relevance. Notably, while individual alterations in these features lacked cancer specificity, simultaneous alterations were uniquely indicative of lung cancer. To comprehensively analyze these features, we developed a scoring model, “IMmunoPhenotypic Analysis for Cancer deTection (IMPACT).” Comprehensive analysis using the five features (IMPACT-5) demonstrated high cancer specificity and biomarker efficacy, as evidenced by the high area under the receiver operating characteristic curve values for lung cancer patients (0.9187, 0.9277, and 0.9363 for stage I NSCLC, stage IV NSCLC, and SCLC patients, respectively), in stark contrast to BLD patients (0.5212). These findings suggest that comprehensive analysis of cancer-associated changes in circulating T cells can effectively detect lung cancer from its early stages, proposing immunophenotypic analysis of circulating T cells as an innovative liquid biopsy-based diagnostic biomarker.

**Supplementary Information:**

The online version contains supplementary material available at 10.1186/s40364-024-00713-7.

**To the editor**

Tumor-immune interactions begin at the earliest stages of tumor development [1, 2]. Tumor immunoediting describes how immune cells shape cancer cells to evade immune surveillance [1, 2]. We noted that this “editing” is bi-directional; immune cells also undergo significant changes while interacting with the tumor. These changes are not confined to the tumor microenvironment but are also evident in peripheral blood [3–8]. These alterations include decreased naïve T cells and increased effector memory T cells in circulation [9]. Cancer-associated cytokines induce phenotypic changes in circulating T cells, such as CXCR3 and LAG3 expression [7, 10]. Based on this, we hypothesized that measuring cancer-associated immune alterations in the circulation could indicate the presence of a tumor, serving as a diagnostic biomarker.

## Study design

**Fig. 1:**
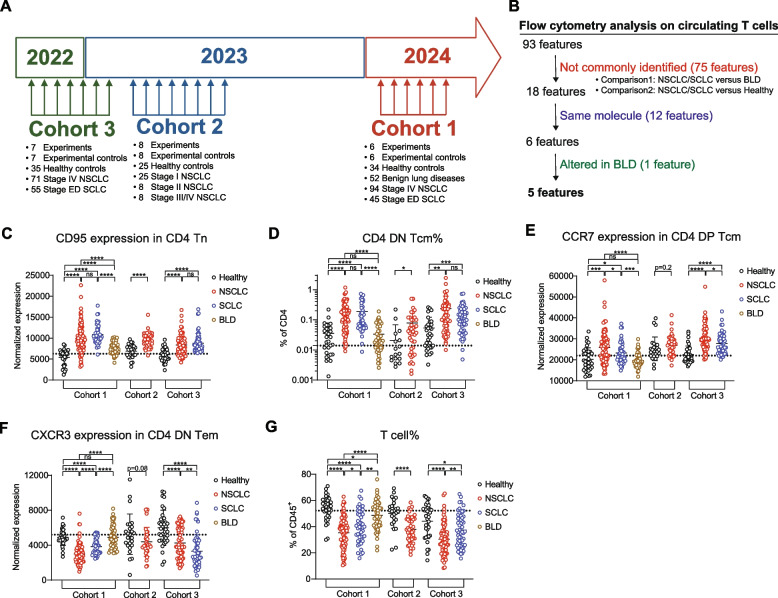
Selection of five T-cell features with diagnostic potential from flow cytometric data of lung cancer patients. **A** Time layout explaining how the experiments were carried out. Flow cytometry analysis was carried out for 3 cohorts comprising 21 independent experiments from late 2022 to early 2024. Details about the samples in each cohort are specified below each cohort. **B** Feature selection protocol. Initial 93 features from the flow cytometric analysis on circulating T cells were applied with three filters. Features that were 1) not commonly identified in two comparisons, 2) derived from the same molecule but differed only in T-cell subsets, or 3) were similarly regulated in BLD patients were excluded. **C**-**G** The five selected features with diagnostic potential. The five features are (**C**) CD95 expression in CD4 Tn, (**D**) CD4 DN Tcm frequency relative to CD4 T cells, (**E**) CCR7 expression in CD4 DP Tcm, (**F**) CXCR3 expression in CD4 DN Tem, and (**G**) T cell frequency relative to CD45^+^ PBMCs. Dotted lines represent the median of healthy controls. Means and standard deviations are shown in the graph. (*n* = 34, 94, 45, 52, 25, 41, 35, 71, and 55 in Cohort 1 Healthy, NSCLC, SCLC, BLD, Cohort 2 Healthy, NSCLC, Cohort 3 Healthy, NSCLC, and SCLC, respectively). Statistical significance was calculated using Student’s t-tests. *p < 0.05, **p < 0.01, ***p < 0.001, ****p < 0.0001. NSCLC, non-small cell lung cancer; SCLC, small cell lung cancer; BLD, benign lung disease; Tn, naïve T cells; Tcm, central memory T cells; Tem, effector memory T cells; DN, CD27 and CD28 double negative; DP, CD27 and CD28 double positive; ns, not significant

To explore this potential, we retrospectively analyzed peripheral blood mononuclear cells (PBMCs) from 206 non-small-cell lung cancer (NSCLC) and 100 small-cell lung cancer (SCLC) patients. PBMCs from 52 patients with benign lung disease (BLD) and 94 healthy individuals were used as non-cancer controls (Table S1). The analysis was performed through 3 independent cohorts (Fig. 1A).

We focused on T cells, given their close interaction with tumors [1, 2]. We identified 18 T-cell subsets using ten phenotypic markers (Fig. S1 and Table S2) [11]. To minimize experimental variability, we aliquoted PBMCs from a single donor, using one vial per experiment as an experimental control. These controls were used to ensure consistent gating of T-cell subsets and to normalize molecular expressions (Fig. S2).

## Feature selection

To identify cancer-associated T-cell alterations, we initially assessed 93 features (Table S3). Subsequently, we aimed to select features that were altered in cancer patients (Fig. 1B). First, using cohort 1 for discovery, we compared cancer patients (NSCLC/SCLC) with BLD patients (Comparison 1) and healthy controls (Comparison 2), selecting 18 features that were commonly altered (Figs. S3A-C). The selected features included two T-cell frequencies (total T cells and CD4 DN Tcm) and 16 molecular expressions. Notably, the molecular expressions were primarily associated with four molecules (CXCR3, CXCR4, CD95, and CCR7), differing only by T-cell subset (Fig. S3D). Since molecular expressions of the same molecule were highly correlated between T-cell subsets, we selected one feature per molecule (Fig. S3D; asterisked). CXCR4 expression was excluded because it was also altered in BLD patients (Fig. S3E). Ultimately, five features were selected to represent cancer-associated T-cell alterations in circulation, which were confirmed through cohorts 2 and 3 (Fig. 1C-G and Figs. S3F-J).

## Achieving cancer specificity

**Fig. 2:**
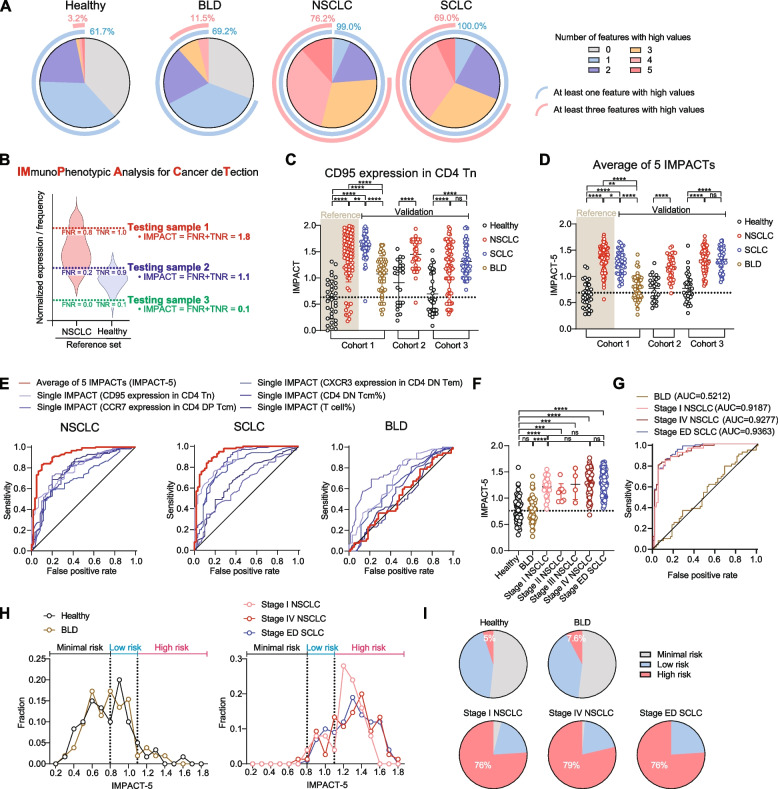
Early detection of lung cancer using comprehensive analysis on cancer-associated T-cell alterations. **A** Pie charts showing the number of features with high values. Values above the median of all samples (including healthy controls and BLD/NSCLC/SCLC patients) were considered high. The proportions of those with at least one (blue) or three (red) features with high values are shown. Features that were downregulated in cancer patients were negated, so that all features appeared upregulated in cancer patients. **B** The IMPACT scoring model. NSCLC patients and healthy controls from cohort 1 were used as the reference set. For each testing sample, its value was used as a threshold to compute the false negative rate (FNR) and true negative rate (TNR). The combined value (FNR + TNR), termed the IMPACT score, ranges from 0 to 2. A higher IMPACT score indicates a higher likelihood of cancer detection in the testing sample. **C** The IMPACT score for one of the five features, CD95 expression in CD4 Tn. Healthy individuals and NSCLC patients from Cohort 1 were used as the reference set. Individuals not included in the reference set are collectively designated as the validation set. Dotted lines represent the median of healthy controls. Means and standard deviations are shown in the graph. (*n* = 34, 94, 45, 52, 25, 41, 35, 71, and 55 in Cohort 1 Healthy, NSCLC, SCLC, BLD, Cohort 2 Healthy, NSCLC, Cohort 3 Healthy, NSCLC, and SCLC, respectively) **D** Average of the 5 IMPACT scores (IMPACT-5) in each group. The dotted line represents the median of healthy controls. Means and standard deviations are shown in the graph. **E** ROC curves of IMPACT scores analyzed individually or comprehensively. The ROC curve was generated for NSCLC (left), SCLC (middle), or BLD (right) patients against healthy controls in the validation set. For each patient group, IMPACT was analyzed individually (5 shades of blue) or comprehensively by averaging the 5 IMPACT scores (red). **F** IMPACT-5 scores for the validation set. The validation set was grouped based on disease status and stages (*n* = 60, 52, 25, 8, 4, 75, and 100 in Healthy, BLD, Stage I NSCLC, Stage II NSCLC, Stage III NSCLC, Stage IV NSCLC, and Stage ED SCLC, respectively). The dotted line represents the median of healthy controls. Means and standard deviations are shown in the graph. **G** ROC curves of IMPACT-5 scores in the validation set. BLD, Stage I NSCLC, Stage IV NSCLC, and Stage ED SCLC patients were analyzed against healthy controls. AUC for each curve is shown. **H** IMPACT-5 distribution in healthy controls and BLD patients (left), and Stage I NSCLC, Stage IV NSCLC, and Stage ED SCLC patients (right). Score bin categories (minimal, low, and high risk) are shown above the graphs. **I** Pie charts showing the proportion of each risk category for patient groups in the validation set. The proportions of high-risk individuals are shown. Statistical significance was calculated using Student’s t-tests. *p < 0.05, **p < 0.01, ***p < 0.001, ****p < 0.0001. NSCLC, non-small cell lung cancer; SCLC, small cell lung cancer; BLD, benign lung disease; FNR, false negative rate; TNR, true negative rate; IMPACT, Immunophenotypic analysis for cancer detection; Tn, naïve T cells; Tcm, central memory T cells; Tem, effector memory T cells; DN, CD27 and CD28 double negative; DP, CD27 and CD28 double positive; ED, extensive disease; ROC, receiver operating characteristic; AUC, area under the curve; ns, not significant

Although the five selected features were altered in cancer patients, determining whether they were exclusively cancer-specific was challenging, as 60–70% of non-cancer controls exhibited elevations in at least one feature (Fig. 2A and Fig. S4). However, a key observation was that the simultaneous elevation of multiple features was specific to cancer patients (Fig. 2A), suggesting that while individual feature changes may lack cancer specificity, the simultaneous alteration of multiple features could provide cancer specificity.

To comprehensively analyze these simultaneous alterations, we developed a scoring model termed “IMmunoPhenotypic Analysis for Cancer deTection (IMPACT)”. This model uses a reference set to calculate the likelihood of an individual having cancer based on each feature (Fig. 2B). All samples outside the reference set were designated as the validation set and used for subsequent analyses. The average score across all five features (IMPACT-5) demonstrated significantly better biomarker performance than any single feature alone and reduced the distinction between BLD patients and healthy controls (Figs. 2C-E and Fig. S5). These findings highlight the enhanced cancer specificity achieved through this comprehensive approach.

## Early detection of lung cancer using the IMPACT-5

Notably, IMPACT-5 demonstrated significant diagnostic potential for lung cancer detection, even at stage I (Fig. 2F and G). When stratifying individuals into minimal, low, and high-risk categories using IMPACT-5, 76% of stage I NSCLC patients fell into the high-risk group, compared to only 5% of healthy controls (Fig. 2H and I). These findings highlight the strong diagnostic potential of IMPACT-5 for the early detection of lung cancer.

Clinical variables had a minor effect on IMPACT-5 scores and were minimal compared to the substantial differences observed between cancer patients and non-cancer individuals (Fig. S6). These findings demonstrate that comprehensive analysis of cancer-associated T-cell alterations can serve as an innovative liquid biopsy-based diagnostic biomarker.

## Supplementary Information

Supplementary Material 1.

## Data Availability

No datasets were generated or analysed during the current study.

## Abbreviations

BLD: Benign lung disease
IMPACT: Immunophenotypic analysis for cancer detection
NSCLC: Non-small cell lung cancer
PBMC: Peripheral blood mononuclear cell
SCLC: Small cell lung cancer
