# Supplementary material for: Immunophenotypic analysis on circulating T cells for early diagnosis of lung cancer
Source: Biomark Res. 2024 Dec 26;12:161. doi: 10.1186/s40364-024-00713-7 (PMC11674110; doi:10.1186/s40364-024-00713-7)
Supplement: Supplementary file 1 — Supplementary Material 1. [file 40364_2024_713_MOESM1_ESM.docx]

**Supplemental Materials and Methods**

**Human samples**

All patient samples were provided by the Biobank of Chonnam National University Hwasun Hospital, a member of the Korea Biobank Network. No patients were excluded due to clinicopathologic criteria such as sex. Histologically confirmed lung cancer patients were categorized into NSCLC and SCLC groups. NSCLC patients were further divided by TNM stages. Patients with non-cancerous BLD were also analyzed. The BLD group included patients with acute diseases, such as bacterial or fungal pneumonia. Additionally, some BLD patients had chronic lung diseases such as tuberculous granuloma or pneumoconiosis. Blood samples from patients were collected from the cephalic veins using BD Vacutainer (BD). Blood from healthy donors was provided by the Korean Red Cross. Blood samples were processed with Lymphoprep (Stemcell Technologies) to obtain PBMCs, which were then frozen at −80°C using 10% dimethyl sulfoxide (Merck) in fetal bovine serum (Gibco).

**Flow cytometry**

Frozen PBMCs were thawed in a 37ºC water bath and washed twice. PBMCs were then stained with fluorochrome-conjugated antibodies along with Ghost Dye Violet 510 Fixable Viability Dye (Cell Signaling Technology) for 30 minutes on ice. Samples were run on a CytoFLEX LX (Beckman Coulter). The antibodies used for the analysis are listed in Table S2.

**Normalization**

Gating of 18 T cell subsets and molecular expression analyses were performed using FlowJo software (Treestar). Three subsets (CD8 SP28 Temra, CD4 DN Tcm, and CD4 Temra) were excluded from further analysis due to their extremely low frequency. For the remaining 15 subsets, molecular expression (experimental values) was assessed across 21 experimental controls. Average expression values for each subset were then calculated. For each experiment, linear regression was performed between the mean expression values of the subsets and their corresponding experimental expression values. This process generated normalization equations for each experiment. Normalized expression values were then calculated by applying the experimental values to their respective normalization equations.

**Feature selection**

Molecular expressions deemed insignificant (e.g., CCR7 expression in Tem) or derived from extremely rare populations were excluded from initial feature selection. For consistency, features that were downregulated in cancer patients were negated, so that all features appeared upregulated in cancer patients. To evaluate the differences between cancer patients and non-cancer individuals, the false positive rate (FPR) at a sensitivity of 0.8 was calculated for each feature. Features with an FPR lower than 0.5 were deemed significant. A correlation matrix to assess the expression correlation between features was generated using the corrplot R package.

**IMPACT scoring model**

The IMPACT score of a test subject was calculated using the reference set. The IMPACT score was determined by adding the negative rate of reference patients (FNR) and the negative rate of healthy controls (TNR), both calculated based on the test subject’s value. The average IMPACT scores of the five features were termed IMPACT-5. Healthy individuals and NSCLC patients from Cohort 1 served as the reference set, while all other individuals were collectively designated as the validation set.

**Statistics**

The statistical significances were tested using Student’s T test. Values of *p < 0.05, **p < 0.01, ***p < 0.001, ****p < 0.0001 were considered significant.


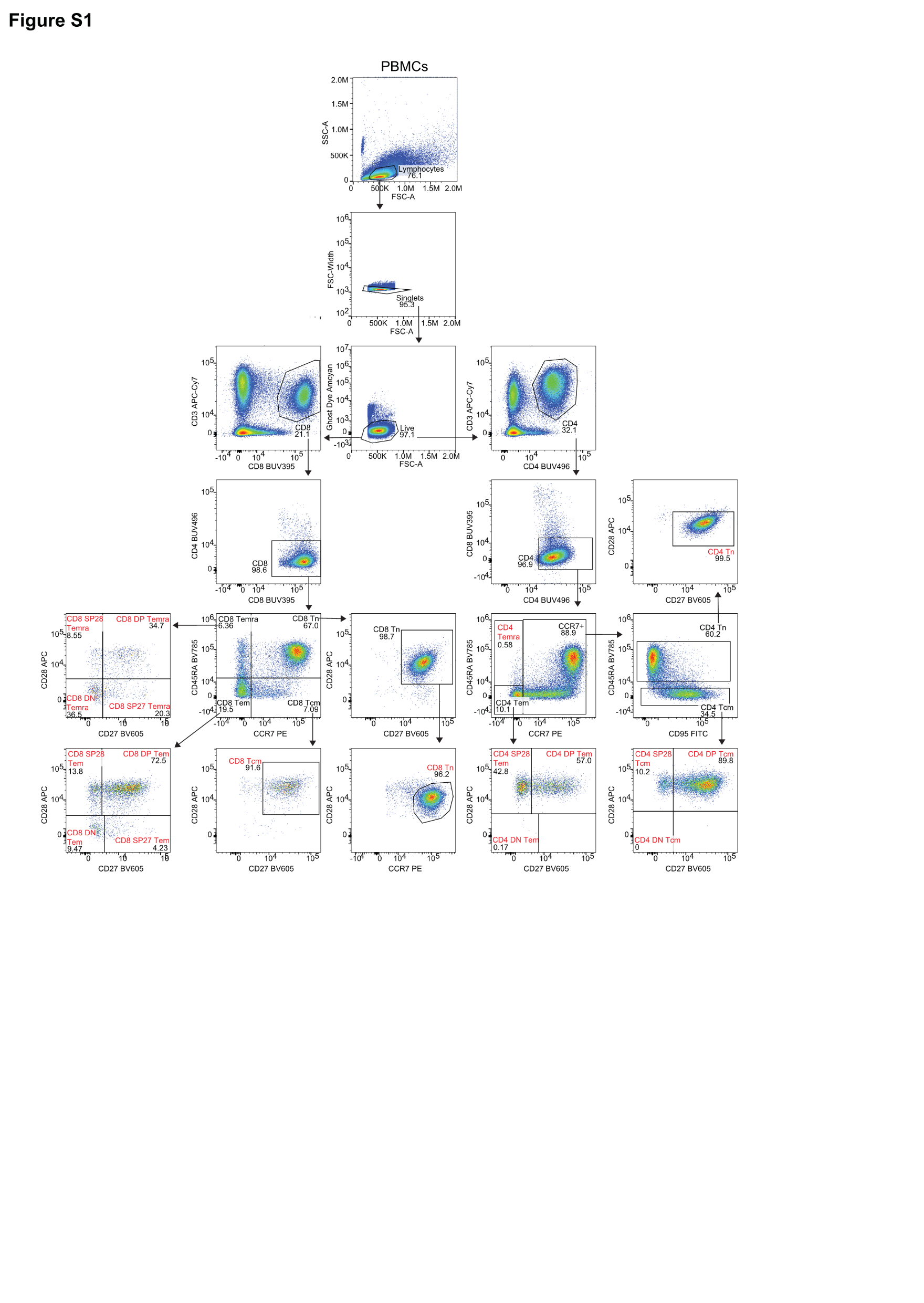


**Fig. S1**

Gating strategy for T cell subsets in the peripheral blood. PBMCs were analyzed using flow cytometry. Total of 18 T cell subsets were gated in experimental controls and applied to other samples. The 18 T cell subsets are: 1. CD8 Tn, 2. CD8 Tcm, 3. CD8 DP Tem, 4. CD8 SP27 Tem, 5. CD8 SP28 Tem, 6. CD8 DN Tem, 7. CD8 DP Temra, 8. CD8 SP27 Temra, 9. CD8 DN Temra, 10. CD4 Tn, 11. CD4 DP Tcm, 12. CD4 SP28 Tcm, 13. CD4 DP Tem, 14. CD4 SP28 Tem, 15. CD4 DN Tem, 16. CD8 SP28 Temra, 17. CD4 DN Tcm, 18. CD4 Temra.

Tn, naïve T cells; Tcm, central memory T cells; Tem, effector memory T cells; Temra, CD45RA re-expressing effector memory T cells; DP, CD27 and CD28 double positive; SP28, CD28 single positive; SP27, CD27 single positive; DN, CD27 and CD28 double negative.


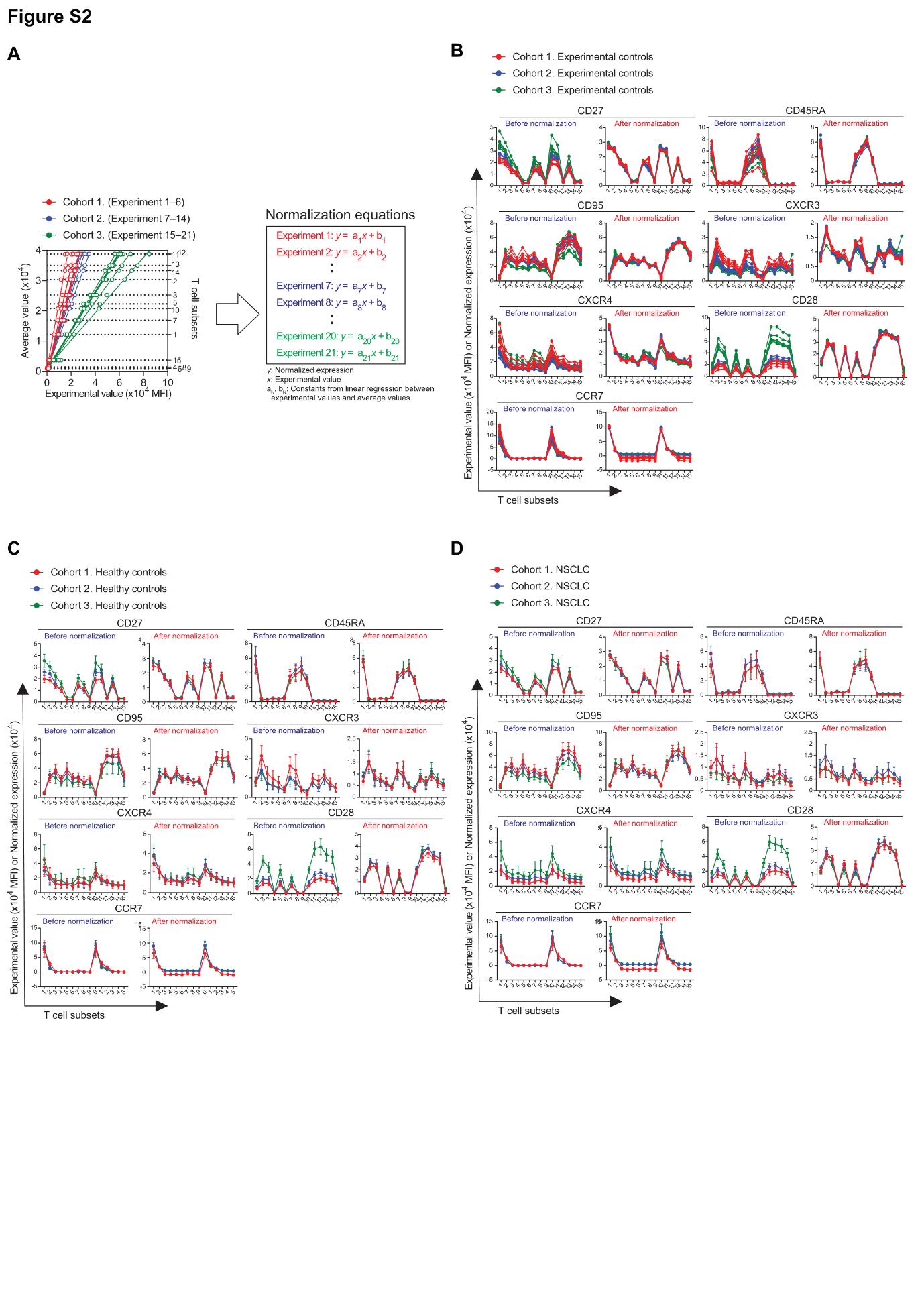


**Fig. S2**

Normalization of molecular expression levels. (A) Method for normalization. For each T cell subset (subsets 1 to 15), the average value of all 21 experimental controls was accessed. Subsequently, linear regression between these average values and experimental values was performed for each experiment. The resulting equation was used for normalization. Dotted lines indicate each T cell subsets. Solid lines represent linear regressions. (B-C) Expression of 7 molecules in 15 different T cell subsets of (B) experimental controls (n=6, 8, and 7 for cohort 1, 2, and 3, respectively), (C) healthy controls (n=34, 35, and 25 for cohort 1, 2, and 3, respectively), and (D) NSCLC patients (n=94, 41, and 71 for cohort 1, 2, and 3, respectively) before and after normalization using normalization equations. Each dot and error bar represent mean and standard deviation.

NSCLC, non-small cell lung cancer.

**
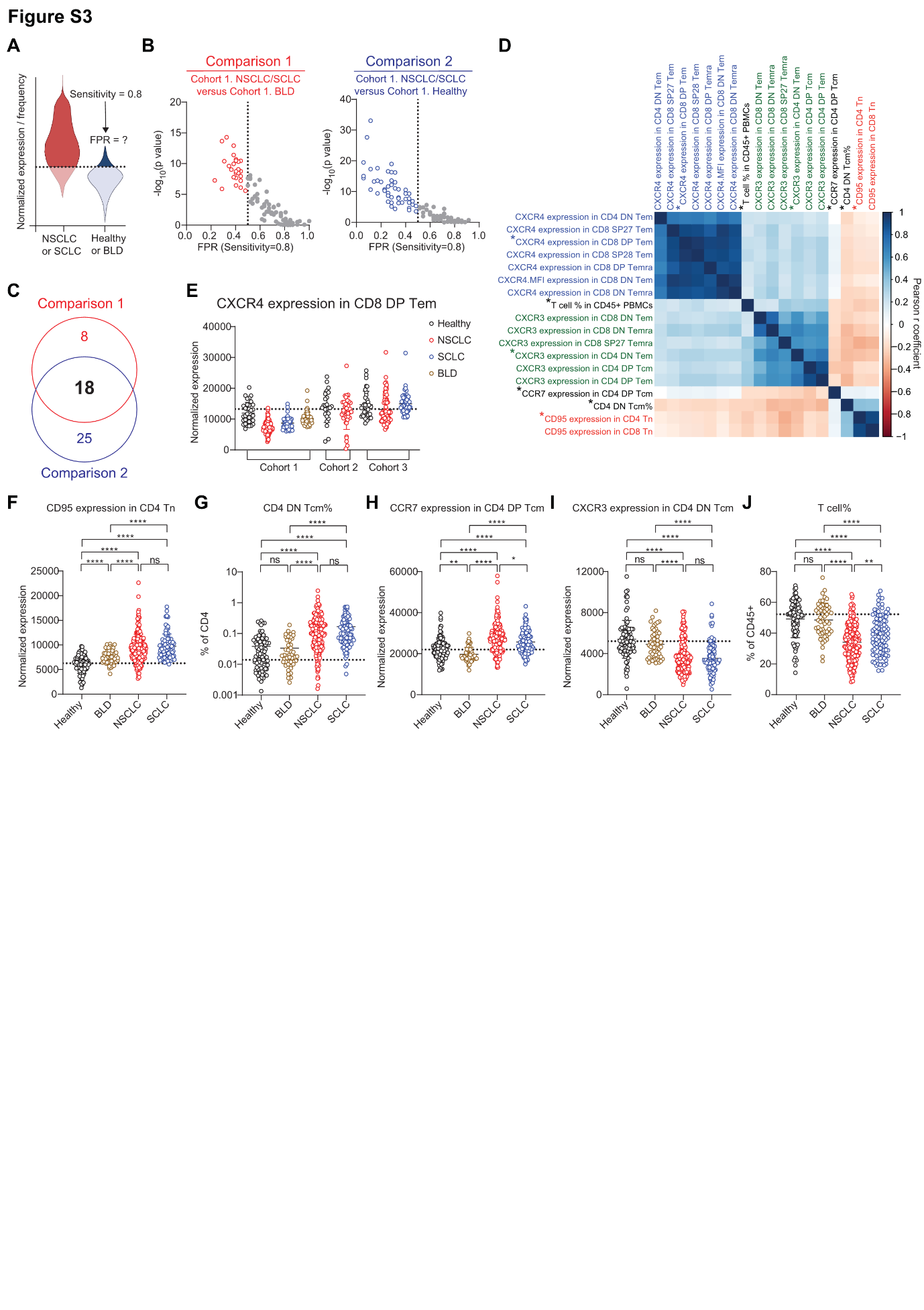
**

**Fig. S3**

Feature selection protocols. (A) Method to extract features with diagnostic potential. For each feature (normalized expressions or T cell subset frequencies), a threshold was set to achieve a sensitivity of 0.8 for NSCLC or SCLC patients. Using this threshold, the FPR was calculated for healthy controls or BLD patients. (B) FPR (at 0.8 sensitivity) and statistical significance [-log_10_(p-value)] for 93 features in two different comparisons. Dotted lines represent the threshold used for feature selection. Features selected for having good biomarker power (FPR < 0.5) are color-coded. (C) Venn diagram showing the overlap of identified features between the two comparisons. 18 features were commonly identified. (D) Correlation matrix for the 18 commonly identified features. Features derived from the same molecule but differing only in T-cell subsets are color-coded (red, blue, and green for CD95, CXCR4, and CXCR3, respectively). The Pearson r coefficients are color-coded from red (low) to blue (high). The selected five features are asterisked. (E) Expression of CXCR4 expression in CD8 DP Tem. Dotted lines represent the median of healthy controls. Means and standard deviations are shown in the graph. (n=34, 94, 45, 52, 25, 41, 35, 71, and 55 in Cohort 1 Healthy, NSCLC, SCLC, BLD, Cohort 2 Healthy, NSCLC, Cohort 3 Healthy, NSCLC, and SCLC, respectively). (F-J) The values of the five selected features in the whole population set (n=94, 52, 206, 100 in Healthy, BLD, NSCLC, and SCLC, respectively). Dotted lines represent the median of healthy controls. Means and standard deviations are shown in the graph.

Statistical significance was calculated using Student’s t-tests. *p < 0.05, **p < 0.01, ***p < 0.001, ****p < 0.0001. NSCLC, non-small cell lung cancer; SCLC, small cell lung cancer; BLD, benign lung disease; FPR, false positive rate; Tn, naïve T cells; Tcm, central memory T cells; Tem, effector memory T cells; DN, CD27 and CD28 double negative; DP, CD27 and CD28 double positive; ns, not significant.


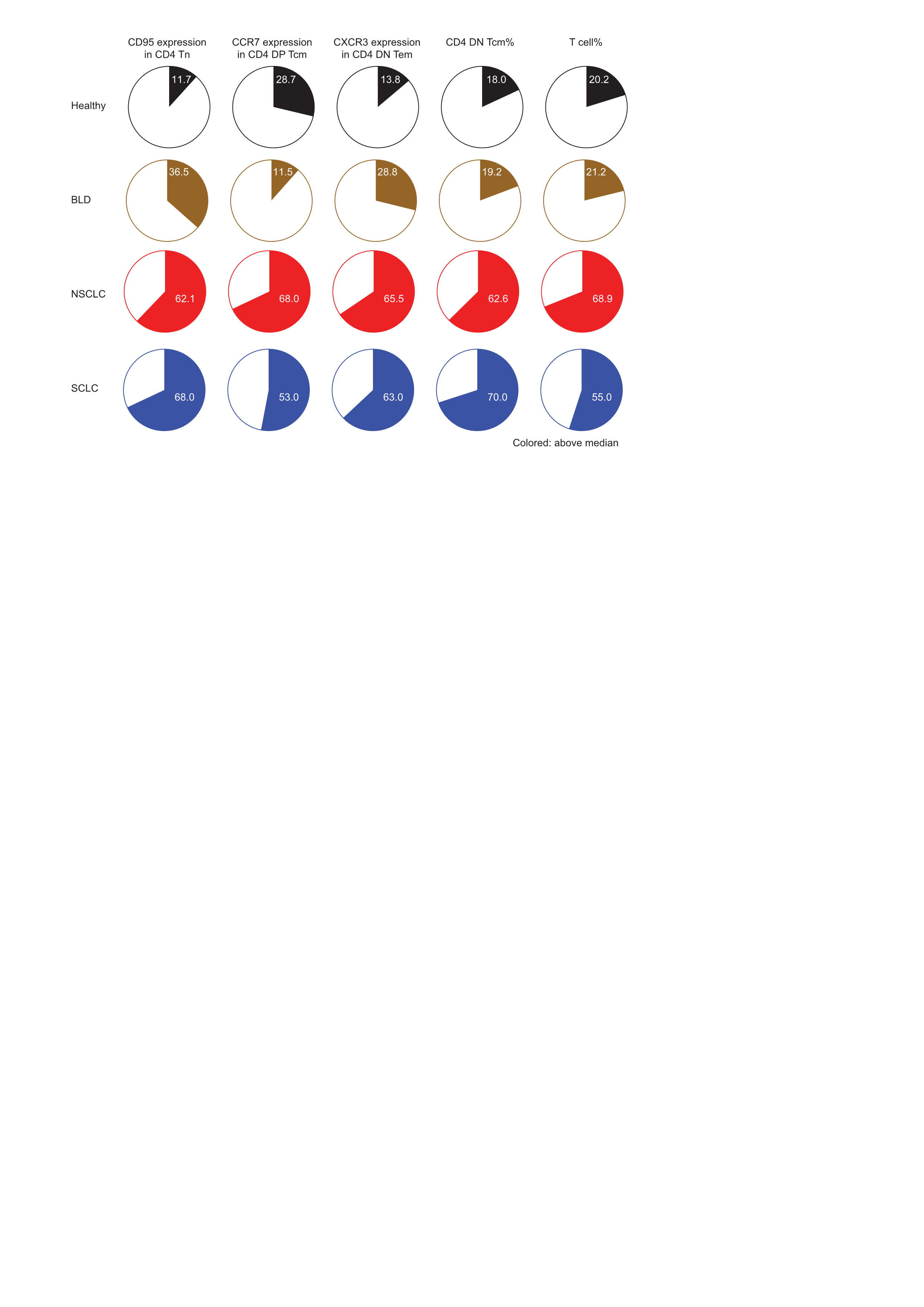


**Fig. S4**

Proportion of elevated values. The proportion of individuals with elevated values (higher than the median of all individuals combined) for each feature were assessed across the cohorts. For consistency, features that were downregulated in cancer patients were negated, so that all features appeared upregulated in cancer patients.

NSCLC, non-small cell lung cancer; SCLC, small cell lung cancer; BLD, benign lung disease; Tn, naïve T cells; Tcm, central memory T cells; Tem, effector memory T cells; DN, CD27 and CD28 double negative; DP, CD27 and CD28 double positive.


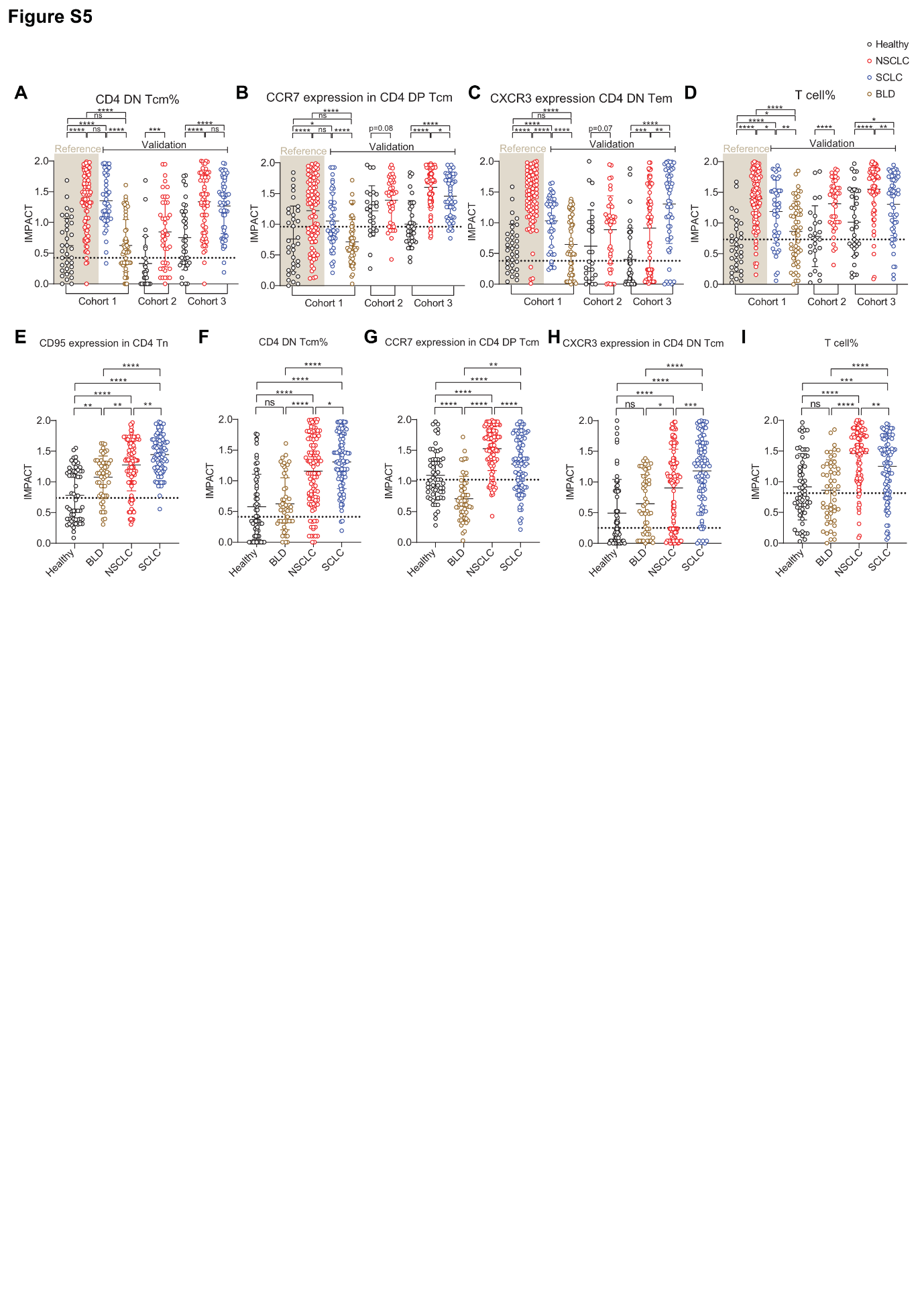


**Fig. S5**

IMPACT score. (A-D) IMPACT scores of the four features (n=34, 94, 45, 52, 25, 41, 35, 71, and 55 in Cohort 1 Healthy, NSCLC, SCLC, BLD, Cohort 2 Healthy, NSCLC, Cohort 3 Healthy, NSCLC, and SCLC, respectively). (E-F) IMPACT scores analyzed in the validation set (n=60, 52, 112, 100, in Healthy, BLD, NSCLC, and SCLC, respectively). Dotted lines represent the median of healthy controls. Means and standard deviations are shown in the graph.

Statistical significance was calculated using Student’s t-tests. *p < 0.05, **p < 0.01, ***p < 0.001, ****p < 0.0001. NSCLC, non-small cell lung cancer; SCLC, small cell lung cancer; BLD, benign lung disease; Tcm, central memory T cells; Tem, effector memory T cells; DN, CD27 and CD28 double negative; DP, CD27 and CD28 double positive; ns, not significant.


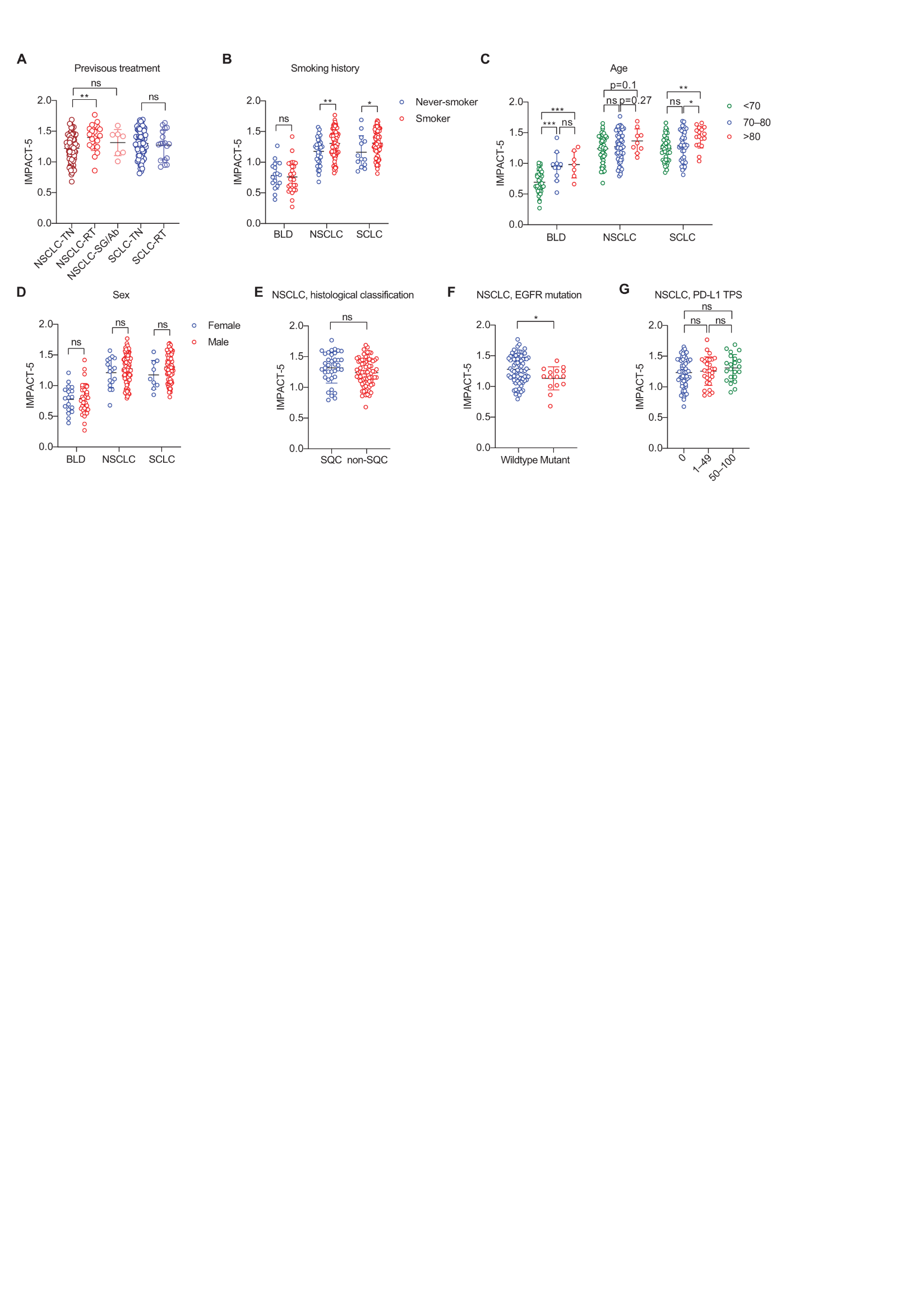


**Fig. S6**

IMPACT-5 scores by clinical variables. (A) NSCLC and SCLC patients were grouped based on their treatment history (treatment-naïve (TN), radiotherapy (RT), or surgery/antibody therapy (SG/Ab)) and assessed for IMPACT-5 scores. (B-D) IMPACT-5 scores were compared among BLD, NSCLC, and SCLC patients based on (B) smoking history, (C) age, and (D) sex. (E-G) NSCLC patients were further analyzed for IMPACT-5 scores by (E) histological classification, (F) EGFR mutation status, and (G) PD-L1 TPS. All analyses were performed within the validation set. Means and standard deviations are shown in the graph.

Statistical significance was determined using Student’s t-tests. *p < 0.05, **p < 0.01, ***p < 0.001. Abbreviations: NSCLC, non-small cell lung cancer; SCLC, small cell lung cancer; BLD, benign lung disease; TN, treatment-naïve; RT, radiotherapy; SG, surgery; Ab, antibody therapy; SQC, squamous cell carcinoma; EGFR, epidermal growth factor receptor; TPS, tumor proportion score; ns, not significant.

|  | Characteristic |  | All cohorts | Cohort 1. | Cohort 2. | Cohort 3. |
| --- | --- | --- | --- | --- | --- | --- |
| NSCLC | Stage | I | 25 | 0 | 25 | 0 |
|  |  | II | 8 | 0 | 8 | 0 |
|  |  | III | 4 | 0 | 4 | 0 |
|  |  | IV | 169 | 94 | 4 | 71 |
|  | Sex | Female | 29 | 12 | 10 | 7 |
|  |  | Male | 177 | 82 | 31 | 64 |
|  | Age | <50 | 5 | 0 | 1 | 4 |
|  |  | 50–60 | 14 | 8 | 3 | 3 |
|  |  | 60–70 | 66 | 31 | 10 | 25 |
|  |  | 70–80 | 97 | 40 | 24 | 33 |
|  |  | >80 | 24 | 15 | 3 | 6 |
|  | Histology | SQC | 78 | 41 | 13 | 24 |
|  |  | Non-SQC | 128 | 53 | 28 | 47 |
|  | Smoking | Never-smoker | 52 | 17 | 21 | 14 |
|  |  | Smoker | 146 | 69 | 20 | 57 |
|  |  | Unknown | 8 | 8 | 0 | 0 |
|  | EGFR | Wildtype | 143 | 70 | 23 | 50 |
|  |  | Mutant | 15 | 0 | 13 | 2 |
|  |  | NA | 48 | 24 | 5 | 19 |
|  | PD-L1 TPS | 0 | 96 | 40 | 23 | 33 |
|  |  | 1–49 | 45 | 17 | 10 | 18 |
|  |  | 50–100 | 52 | 30 | 6 | 16 |
|  |  | NA | 13 | 7 | 2 | 4 |
|  | Pretreatments | None | 148 | 68 | 37 | 43 |
|  |  | Radiotherapy | 46 | 25 | 1 | 20 |
|  |  | Surgery | 6 | 1 | 2 | 3 |
|  |  | Antibody therapy | 2 | 0 | 1 | 1 |
|  |  | NA | 4 | 0 | 0 | 4 |
| SCLC | Stage | ED | 100 | 45 |  | 55 |
|  | Sex | Female | 9 | 6 |  | 3 |
|  |  | Male | 91 | 39 |  | 52 |
|  | Age | <50 | 0 | 0 |  | 0 |
|  |  | 50–60 | 7 | 6 |  | 1 |
|  |  | 60–70 | 35 | 16 |  | 19 |
|  |  | 70–80 | 41 | 16 |  | 25 |
|  |  | >80 | 17 | 7 |  | 10 |
|  | Smoking | Never-smoker | 13 | 6 |  | 7 |
|  |  | Smoker | 85 | 37 |  | 48 |
|  |  | Unknown | 2 | 2 |  | 0 |
|  | Pretreatments | None | 79 | 35 |  | 44 |
|  |  | Radiotherapy | 18 | 10 |  | 8 |
|  |  | Surgery | 0 | 0 |  | 0 |
|  |  | Antibody therapy | 0 | 0 |  | 0 |
|  |  | NA | 3 | 0 |  | 3 |
| BLD | Disease state | Acute | 31 | 31 |  |  |
|  |  | Chronic | 21 | 21 |  |  |
|  | Sex | Female | 18 | 18 |  |  |
|  |  | Male | 34 | 34 |  |  |
|  | Age | <50 | 4 | 4 |  |  |
|  |  | 50–60 | 6 | 6 |  |  |
|  |  | 60–70 | 24 | 24 |  |  |
|  |  | 70–80 | 11 | 11 |  |  |
|  |  | >80 | 7 | 7 |  |  |
|  | Smoking | Never-smoker | 17 | 17 |  |  |
|  |  | Smoker | 27 | 27 |  |  |
|  |  | Unknown | 8 | 8 |  |  |

**Table S1. Patient information**

A total of 206 NSCLC patients, 100 SCLC patients, and 52 BLD patients were analyzed across three independent cohorts. Information about these patients, both as a combined group (All cohorts) and individually for each cohort (Cohort 1, Cohort 2, and Cohort 3), is presented.

NSCLC, non-small cell lung cancer; SCLC, small cell lung cancer; BLD, benign lung disease; SQC, squamous cell carcinoma; ED, extensive disease; EGFR, epidermal growth factor receptor; TPS, tumor proportion score; NA, not available.

| **Antibody** | **Clone** | **Fluorochromes for Cohort 1/2** | **Fluorochromes for Cohort 3** |
| --- | --- | --- | --- |
| Anti-human CCR7 | G043H7 | PE  (BioLegend, 353204) | PE  (BioLegend, 353204) |
| Anti-human CD27 | O323 or  M-T271 | BV605  (BioLegend, 353204) | BUV496  (BD, 741145) |
| Anti-human CD28 | CD28.2 | APC  (BioLegend, 302912) | BUV737  (BD, 612815) |
| Anti-human CD3 | UCHT1 | APC-Cy7  (BioLegend, 300426) | APC-Cy7  (BioLegend, 300426) |
| Anti-human CD4 | RPA-T4 or  OKT4 | BUV496  (BD, 741134) | BV785  (BioLegend, 317442) |
| Anti-human CD45RA | HI100 | BV785  (BioLegend, 304140) | BV605  (BioLegend, 304134) |
| Anti-human CD8 | RPA-T8  SK1 | BUV395  (BD, 563795) | FITC  (BioLegend, 344704) |
| Anti-human CD95 | DX2 | FITC  (Invitrogen, 2785780) | BUV395  (BD, 740306) |
| Anti-human CXCR3 | G025H7 | PE-Cy7  (BioLegend, 353720) | PE-Cy7  (BioLegend, 353720) |
| Anti-human CXCR4 | 12G5 | BV421  (BioLegend, 306518) | BV421  (BioLegend, 306518) |

**Table S2. Information about antibodies used for flow cytometry analysis**

The antibodies and their clones used for flow cytometry are listed. The fluorochrome used for each molecule was consistent across Cohorts 1 and 2, while a different set of fluorochromes was used for Cohort 3.

CXCR, CXC motif chemokine receptor; FITC, fluorescein; PE, phycoerythrin; APC, allophycocyanin; BV, brilliant violet; BUV, brilliant ultra violet.

| **T cell subset No.** | **Subset name** | **Frequency** | **CD95** | **CXCR3** | **CXCR4** | **CD27** | **CD28** | **CCR7** | **CD45RA** |
| --- | --- | --- | --- | --- | --- | --- | --- | --- | --- |
| **1** | CD8 Tn | O | O | O | O | O | O | O | O |
| **2** | CD8 Tcm | O | O | O | O | O | O | O | X |
| **3** | CD8 DP Tem | O | O | O | O | O | O | X | X |
| **4** | CD8 SP27 Tem | O | O | O | O | O | X | X | X |
| **5** | CD8 SP28 Tem | O | O | O | O | X | O | X | X |
| **6** | CD8 DN Tem | O | O | O | O | X | X | X | X |
| **7** | CD8 DP Temra | O | O | O | O | O | O | X | O |
| **8** | CD8 SP27 Temra | O | O | O | O | O | X | X | O |
| **9** | CD8 DN Temra | O | O | O | O | X | X | X | O |
| **10** | CD4 Naïve | O | O | O | O | O | O | O | O |
| **11** | CD4 DP Tcm | O | O | O | O | O | O | O | X |
| **12** | CD4 SP28 Tcm | O | O | O | O | X | O | O | X |
| **13** | CD4 DP Tem | O | O | O | O | O | O | X | X |
| **14** | CD4 SP28 Tem | O | O | O | O | X | O | X | X |
| **15** | CD4 DN Tem | O | O | O | O | X | X | X | X |
| **16** | CD8 SP28 Temra | O | X | X | X | X | X | X | X |
| **17** | CD4 DN Tcm | O | X | X | X | X | X | X | X |
| **18** | CD4 Temra | O | X | X | X | X | X | X | X |

**Table S3. Information about candidate features**

PBMCs were gated into 18 different T cell subsets. The frequency of all 18 T cell subsets (relative to either CD8 T cells or CD4 T cells) was considered as candidate features. Additionally, the molecular expression of 7 surface markers within these 18 subsets was also considered as potential candidate features. However, expressions that were negligibly expressed or those in subsets with extremely low frequency (T cell subsets 16, 17, and 18), which could yield unreliable expression data, were excluded. Candidate features and excluded features are indicated as O and X, respectively. In addition, frequency of total T cells relative to CD45^+^ PBMCs was considered as a candidate feature.

PBMC, peripheral blood mononuclear cells; Tn, naïve T cells; Tcm, central memory T cells; Tem, effector memory T cells; DN, CD27 and CD28 double negative; DP, CD27 and CD28 double positive; CXCR, CXC motif chemokine receptor.
